# Supplementary material for: Laser harmonic generation with independent control of frequency and orbital angular momentum
Source: Nat Commun. 2024 Aug 11;15:6878. doi: 10.1038/s41467-024-51311-y (PMC11317519; doi:10.1038/s41467-024-51311-y)
Supplement: Supplementary file 1 — Supplementary Information [file 41467_2024_51311_MOESM1_ESM.pdf]

Supplemental Material to "Laser harmonic generation with independent control of frequency and orbital angular momentum"

R. M. G. M. Trines,[1] H. Schmitz,[1] M. King,[2,3] P. McKenna,[2,3] and R. Bingham[1,2]

[1] *Central Laser Facility, STFC Rutherford Appleton Laboratory, Didcot, OX11 0QX, United Kingdom*
[2] *SUPA, Department of Physics, University of Strathclyde, Glasgow, G4 0NG, United Kingdom*
[3] *The Cockcroft Institute, Sci-Tech Daresbury, Warrington WA4 4AD, United Kingdom*

(Dated: June 24, 2024)

S1. DIAGNOSING THE HARMONIC SPECTRUM FROM SIMULATIONS

In this section, we expand upon the technique used throughout the main text to diagnose the harmonic spectrum obtained from our 3D simulations. We are obliged to study the electric field $\vec{E}$, since our simulation results do not include the vector potential $\vec{A}$.

A transverse real-valued electric field $\vec{E}_\perp$ can be decomposed into two orthogonal linear polarisations: $\vec{E}_\perp = (\vec{E}_\perp \cdot \vec{e}_x)\vec{e}_x + (\vec{E}_\perp \cdot \vec{e}_y)\vec{e}_y$, where $\vec{E}_\perp \cdot \vec{e}_x$ and $\vec{E}_\perp \cdot \vec{e}_y$ are independent real-valued quantities. Alternatively, $\vec{E}_\perp$ can also be decomposed into two orthogonal circular polarisations: $\vec{E}_\perp = E_+\vec{e}_+ + E_-\vec{e}_-$, with $\vec{e}_\pm = (\vec{e}_x \pm i\vec{e}_y)/\sqrt{2}$ and $E_\pm = \vec{E}_\perp \cdot \vec{e}_\pm$ complex-valued. However, since $\vec{E}_\perp^* = \vec{E}_\perp$ while $\vec{e}_-^* = \vec{e}_+$, we find that $E_- = E_+^*$. In the frequency domain, we find that the Fourier transform $\mathcal{F}[E_-](\omega) = (\mathcal{F}[E_+](-\omega))^*$, i.e. the RCP frequency spectrum for $\omega > 0$ is the complex conjugate of the LCP frequency spectrum for $\omega < 0$ and vice versa. This motivates us to only use the E_+ coefficient, or actually $\tilde{\Psi} \equiv E_x + iE_y = E_+\sqrt{2} = -\partial\Psi/\partial t$, and to use the "signed frequency" $-\infty < \omega/\sigma < \infty$, as in Section S2. Thus, $\mathcal{F}[\tilde{\Psi}](\omega/\sigma)$ captures all the spectral information of $\vec{E}_\perp$, while each value of ω/σ represents a mode with pure circular polarisation. Naturally, the use of ω/σ in the Fourier domain implies the use of $\vec{k}/\sigma$, ℓ/σ, etc. also.

Finally, we note that a harmonic progression in $(\omega/\sigma, \vec{k}/\sigma)$ space, driven by Eqns. (E2)-(E6) will always be regular, i.e. regularly spaced peaks on a 1-D line or a 2-D grid, unlike a harmonic progression in $(\omega, \vec{k})$ space [1, 2].

This technique is especially important in the case of a circularly polarised pump and a harmonic with opposite spin to the pump, since such harmonics tend to be drowned out by their counterpart with the same spin as the pump. See, for example, Li *et al.* [3], in which harmonics with opposite spin are predicted but not observed, as their amplitude is assumed to be "too small" to be detected easily.

S2. CONVENTIONS

In the main text, we study laser harmonic generation (HG) by a cubic nonlinear term $|\vec{A}|^2\vec{A}$ in the wave equation for the vector potential $\vec{A}$ of the electromagnetic (laser) field. This covers the most common scenarios for harmonic generation. Throughout, we use the following conventions:

1. We write all laser fields as superpositions of circularly polarised, CP, laser modes. For example, a laser pulse with linear (elliptic) polarisation is the superposition of *two* CP modes with equal (unequal) amplitudes and opposite spin.

2. We assume a plane electromagnetic wave travelling in the z-direction and use the function $\Psi = A_x + iA_y$ to describe the transverse EM fields, where (A_x, A_y) represents the transverse vector potential. For a CP vector potential $(\cos(\omega t - \ell\varphi), (1/\sigma)\sin(\omega t - \ell\varphi))$, where $\sigma = \pm 1$ denotes the spin direction, we find that $\Psi = \exp[i(\omega t - \ell\varphi)/\sigma]$. For two such modes $\vec{A}_A$ and $\vec{A}_B$, we find that $\vec{A}_A \cdot \vec{A}_B = \Re(\Psi_B^*\Psi_A) \propto \cos[(\omega_B/\sigma_B - \omega_A/\sigma_A)t - (\ell_B/\sigma_B - \ell_A/\sigma_A)\varphi]$. The nonlinear term in the wave equation for Ψ then becomes $(\Psi^*\Psi)\Psi$. This convention will also allow us to make use of the complex Mathieu equation [4].

3. In light of the above, we use the quotients ω/σ, $\vec{k}/\sigma$, ℓ/σ, etc. instead of σ and ω separately. While ω/σ can be any real number, we always use $\omega > 0$, which uniquely defines σ. This convention renders both the calculation and the visualisation of harmonic progressions easier and more intuitive. We use ω/σ rather than $\sigma\omega$ since $\omega/\sigma = \hbar\omega/(\hbar\sigma) = \mathcal{E}/S_z$, with $\mathcal{E}$ and S_z denoting the photon energy and spin. The quantity $\sigma\omega$ does not have such an elegant physical interpretation.

4. We treat left and right CP modes at the same harmonic frequency ω as separate harmonics with opposite signs of ω/σ rather than different polarisations of the same harmonic. We do this so that we can treat the spin σ on a footing equal to the frequency ω, wave vector $\vec{k}$ or OAM level ℓ. For example, sending a left CP laser beam through a half-wave plate to create a right CP beam will be treated as generating harmonic -1, since $\omega/\sigma \to -\omega/\sigma$ in that case.

5. In terms of the harmonic progression, we treat harmonic cascades in quantities like ω, $k_\perp$ or ℓ on an

equal footing as much as possible. So a cascade in ℓ at a single frequency ω_0 [5] is treated as a harmonic progression just like a cascade in ω at the single OAM level $\ell = 0$. This way, multi-dimensional cascades in, for example, $(\omega/\sigma, \ell/\sigma)$ space are also possible.

The justification for these conventions is as follows:

(i) A CP wave has constant amplitude A^2 and does not beat with itself (unlike an LP wave), while two CP waves with parallel $\vec{k}$ beat in precisely one way.

(ii) A CP wave is a spin eigenstate, while LP and EP waves are not. Thus, CP waves can be used to verify the spin conservation law, while LP waves cannot be used for that.

(iii) Since a CP wave has constant A^2, it obeys all possible translation/rotation symmetries. Adding a second CP wave will break the symmetry of A^2 in one specific way, leading to harmonic generation in one specific dimension in $(\omega/\sigma, \vec{k}/\sigma)$ space. This relates the dimensionality of symmetry breaking to the dimensionality of the harmonic progression.

Thus, CP waves are more fundamental "building blocks" to study HG than LP waves. Using the above conventions renders it easier to calculate and visualise the harmonic progression. The harmonic progression becomes a "beat wave" process [6, 7]: the beating fixes the harmonic step to $\omega_B/\sigma_B - \omega_A/\sigma_A$ or $\ell_B/\sigma_B - \ell_A/\sigma_A$ etc., while ω/σ (or ℓ/σ etc.) can assume any positive or negative value. It is easy to extend this procedure to more variables ($k_\perp$, ℓ, etc.) or to more CP modes. The harmonic spectrum generated by two CP modes will be a sequence of equidistant points on a line; for three CP modes that are not on the same line in 2-D Fourier space, the generated harmonic spectrum will be a 2-D grid; for four CP modes that are not on the same plane in 3-D Fourier space, the generated harmonic spectrum will be a 3-D lattice.

S3. HARMONIC PROGRESSION

The details of the harmonic progression for HG in low-order nonlinear systems are outlined in this section. In most practical cases, the lowest-order nonlinear term in the wave propagation equation is either cubic, e.g. $(\Psi^*\Psi)\Psi$, or quadratic, e.g. $2(\Psi^*_{DC}\Psi)\Psi$ or similar, where Ψ_{DC} is constant in time. In general, the cubic case is seen in HG in isotropic media where no energy or momentum is left behind in the medium, so energy and momentum are conserved in the EM waves alone. The quadratic case is often seen in HG in anisotropic media where linear or angular momentum (or even energy, as in stimulated Raman scattering) can be left behind in the medium. In the quadratic case, the nonlinear terms can often be rewritten as $|\Psi+\Psi_{DC}|^2\Psi$ or even $|\Psi+\Psi_{DC}|^2(\Psi+\Psi_{DC})$, effectively reducing it to a cubic case providing a joint description of the laser and the medium, see also Ref. [8]. For this reason, we first study the case of a wave equation with cubic nonlinearity:

$$(\partial_t^2 - c^2\nabla^2)\Psi = -(\Omega^2 + \Gamma^2\Psi^*\Psi)\Psi. \quad \text{(E1)}$$

We use a pump beam $\Psi_0 = \Psi_A + \Psi_B + \Psi_C + \ldots$, where $\Psi_A = a_A \exp[i(\omega_A t - k_A z - \ell_A\varphi - k_{\perp,A}x_\perp - \delta_A)/\sigma_A]$, etc. For a single mode $\Psi_0 = \Psi_A$, we find that $\Psi_0^*\Psi_0$ is constant, and no harmonics will be generated. For two modes, $\Psi_0 = \Psi_A + \Psi_B$, we can use the theory of the Mathieu equation and the Jacobi-Anger expansion to find the harmonic frequency progression:

$$\omega_n/\sigma_n = \omega_A/\sigma_A + n(\omega_B/\sigma_B - \omega_A/\sigma_A), \quad n \in \mathbb{Z}. \quad \text{(E2)}$$

Since $\omega_n > 0$ always, σ_n is well-defined. We note that all the frequencies in the ω/σ spectrum are equidistant, while this need not be true at all for the ω spectrum.

Now that σ_n has been determined, we introduce the other harmonic progressions ($n \in \mathbb{Z}$):

$$\ell_n/\sigma_n = \ell_A/\sigma_A + n(\ell_B/\sigma_B - \ell_A/\sigma_A), \quad \text{(E3)}$$

$$k_{\perp,n}/\sigma_n = k_{\perp,A}/\sigma_A + n(k_{\perp,B}/\sigma_B - k_{\perp,A}/\sigma_A), \quad \text{(E4)}$$

$$\delta_n/\sigma_n = \delta_A/\sigma_A + n(\delta_B/\sigma_B - \delta_A/\sigma_A - \arg(\Gamma^2)), \quad \text{(E5)}$$

$$\sigma_n/\sigma_n = \sigma_A/\sigma_A + n(\sigma_B/\sigma_B - \sigma_A/\sigma_A), \quad \text{(E6)}$$

where the last equation is of course trivially true, and ensures that every harmonic step is spin-neutral.

We define $\#_A$, $\#_B$, etc. as the number of photons absorbed from mode A, B, etc. for the generation of mode n, and make use of the fact that any "fundamental mode" is an eigenfunction for energy with eigenvalue $\hbar\omega_A$ etc. Since the nonlinear factor $\Psi^*\Psi$ in (E1) obeys the same symmetries as the wave energy $\propto E^2$, it is safe to assume energy conservation [9–12], i.e. $\omega_n = \#_A\omega_A + \#_B\omega_B$. Then we find:

$$(\#_A, \#_B)_n = \sigma_n[(1/\sigma_A, 0) + n(-1/\sigma_A, 1/\sigma_B)]. \quad \text{(E7)}$$

We note that every quantity that scales in the same way as ω_n, i.e. σ_n, ℓ_n, and $k_{\perp,n}$, will be automatically conserved when ω_n is conserved, due to the form of the expressions for $\#_A$ and $\#_B$, and due to the fact that Ψ_A is an eigenfunction for all these quantities. Due to our conventions, we can calculate $\#_A$ and $\#_B$ explicitly, and get conservation laws for spin, orbital angular momentum and transverse momentum at the same time.

The harmonic progression (E2)-(E6) is characterised by one index n and is essentially one-dimensional. However, with three independent CP modes, a two-dimensional progression in two variables, e.g. ω and ℓ, can be realized. The harmonic progression then becomes:

$$\begin{aligned}\omega_{nm}/\sigma_{nm} = \omega_A/\sigma_A &+ n(\omega_B/\sigma_B - \omega_A/\sigma_A)\\ &+ m(\omega_C/\sigma_C - \omega_A/\sigma_A),\end{aligned} \quad \text{(E8)}$$

$$\begin{aligned}\ell_{nm}/\sigma_{nm} = \ell_A/\sigma_A &+ n(\ell_B/\sigma_B - \ell_A/\sigma_A)\\ &+ m(\ell_C/\sigma_C - \ell_A/\sigma_A).\end{aligned} \quad \text{(E9)}$$

The most visible difference between the 1-D and 2-D progressions is that in a 1-D progression there will always be exactly one OAM level for a given harmonic frequency, while in a 2-D progression there can often be many OAM levels for a given harmonic frequency.

S4. MOVING WINDOW

In order to observe the 2D harmonic spectrum after the laser pulse has propagated into the far-field after interaction with the aperture targets, additional simulations using a moving window were conducted. These were defined with a larger simulation domain of $-20\ \mu\mathrm{m} \leq x, y \leq 20\ \mu\mathrm{m}$ and $-25\ \mu\mathrm{m} \leq z \leq -5\ \mu\mathrm{m}$ with $1440\times1440\times1500$ grid cells. The target and laser parameters were the same as the previous simulations with the exception that the temporal profile was reduced to 30 fs FWHM to ensure the entire pulse fit in the simulation domain. The simulation allowed the laser pulse to propagate for 60 fs before the simulation domain begins to move in z-direction with a velocity of c. This allows the entire pulse to be observed as it moves through the aperture and beyond without having to continually simulate the plasma. The pulse was then sampled when the simulation domain was centred at z=250 μm. Due to the nature of the simulation and as the pulse is now in free-space, a spatial Fourier transform is used instead of temporal and the 2D harmonic spectrum is constructed. This can be seen in Supplementary figure S1**a** and **b** for n=0 and n=3, respectively. As can be observed, the harmonic spectra maintain the same profile, albeit with more numerical noise potentially due to numerical dispersion when considering the pulse in the spatial domain instead of frequency. To ensure we are indeed in the far-field, the spatial profile of the -ω and +ω components of the generated $2\omega_0$ harmonic light are shown in Supplementary figure S1**c** and **d** for the n=0 case and Supplementary figure S1**e** and **f** for the n=3 case, respectively. As can be seen, these correspond to the constructed k-space patterns observed in figure 3**e-h** of the main text.

S5. TARGET STRUCTURE SIZE EFFECTS

The variation of the radius and corrugation depth of the targets was investigated for the transmissive cases. Supplementary figures S2 **a** and **b** show the generated 2D harmonic spectra for an aperture radius of 1.5 μm and corrugation depths of 0.5 μm and 1.5 μm, respectively. The overall structure of the spectra is relatively unchanged, albeit the strengths of the different harmonic peaks vary. The same can be seen in Supplementary figures S2**c-d** for the same corrugation depths, but an increased radius of 2.5 μm. The change in strength of the harmonics can impact on the overall spatial profile of the generated harmonics as shown in the full $2\omega_0$ 2D profile shown Supplementary figures S2**e-h** for the same preceding cases, respectively.

S6. FROM PLASMA PHYSICS TO NONLINEAR OPTICS

Our approach provides access to a vast landscape of extensions, both in laser-plasma interactions and in nonlinear optics. Within plasma physics we find:

(i) Adding OAM to the pump laser, changes the shape of the 2-D grid and thus the frequencies in the comb.

(ii) A tilted target adds a DC mode with $\ell/\sigma = 0$, while more complex targets can add multiple values of ℓ/σ.

(iii) Qu, Jia and Fisch [18] studied a plasma q-plate for $q = 1$ using the magnetic field of two Helmholtz coils. We can now study plasma q-plates for $q \leq 0$ using the magnetic field of a magnetic $2(1 - q)$ multipole (as used for particle beam optics), which already has the required shape.

(iv) By adding OAM to the pump laser, the concept of the non-linear q-plate [19, 20] can be extended to that of the non-linear J-plate [21, 22], provided we use ℓ/σ of both the pump laser and the target as two independent degrees of freedom.

Since HG in nonlinear optics is usually governed by the same cubic nonlinearities, many of our findings can be applied to this field without much modification. Examples include:

(i) The work by Hickstein *et al.* [23], which reports on HG by CP beams (two fundamental modes, 1-D expansion, one transverse angle per frequency) versus LP beams (four modes, 2-D expansion, multiple transverse angles per frequency). The findings of that work can be explained easily using our approach.

(ii) The frequency comb generated by a "necklace beam", as introduced in Rego *et al.* [24]. The two LP beams used can be decomposed into four CP modes with $(\omega/\sigma, \ell/\sigma) = \pm(\omega_0, \ell_1)$ and $\pm(\omega_0, -\ell_2)$. These modes generate a 2-D grid in $(\omega/\sigma, \ell/\sigma)$ space. Selecting the modes with $\ell = 0$ from this grid yields the same frequency comb as reported in Rego *et al.*

(iii) The "torus-knot angular momentum", introduced in Pisanty *et al.* [25] and Dorney *et al.* [26]: the two fundamental modes generate a sequence of harmonic modes that are all on the same line in $(\omega/\sigma, \ell/\sigma)$ space, at regular intervals, without the need to introduce new physical quantities.

(iv) In stimulated Raman scattering [27, 28] or beat wave generation [6, 7], a laser beam interacts with a moving structure in a nonlinear medium to produce side bands. This can be studied using the same approach as HG, including extension from frequency to OAM side bands [5].

These examples illustrate how general our new approach is, and the breadth of its field of applications.

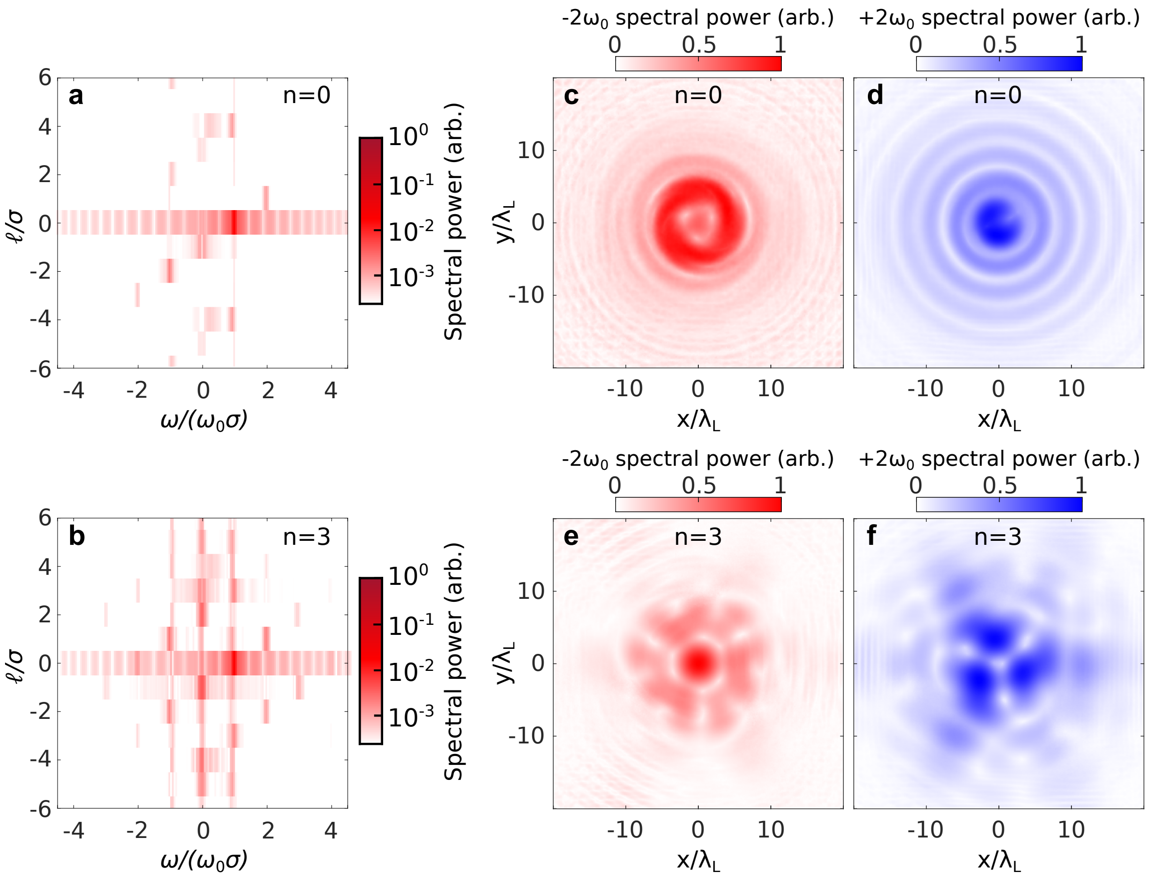

Figure S1: **a-b**, 2D harmonic spectrum for the, **a**, 0-fold and, **b**, 3-fold apertures from the moving window simulation. **c-d**, Spatial profile of the generated -2ω_0 and +2ω_0 light from the circular (0-fold) aperture, respectively. **e-f**, Same for the structured (3-fold) aperture. These were measured when the simulation domain was centred on z=250λ_L.

S7. HEIGHT FUNCTION

In this section, we move beyond the Lichters model [13] and study HG by a laser pulse incident on an arbitrary surface of an opaque target. To describe the target surface, we use a height function $h(x, y)$ and define $\vec{a}_{DC} = \nabla_\perp h$ or $\Psi_{DC} = \partial h/\partial x + i\partial h/\partial y$. For oblique incidence on a plane target this approach yields $h(x, y) = x \tan\alpha$ and $\Psi_{DC} = \tan\alpha$. For more intricate surface shapes, there are almost unlimited possibilities for Ψ_{DC}.

Wang *et al.* [14] reports on the interaction of a circularly polarised laser beam, $\sigma = +1$, with a conically shaped dent in the target surface, half angle α. Li *et al.* [3] reports on the interaction of a tightly focused laser pulse having a concave phase front with a planar target, which is sufficiently similar to Ref. [14] for our purposes. The height function for a conical dent is $h(x, y) = \tan(\pi/2 - \alpha)\sqrt{x^2 + y^2}$ so $\Psi_{DC} = \tan(\pi/2 - \alpha)\exp(i\varphi)$, where φ is the azimuthal angle in polar coordinates. Then the DC mode has $\omega/\sigma = 0$ and $\ell/\sigma = -1$. If this dent is hit by a circularly polarised laser beam with spin σ_0, frequency ω_0 and OAM level ℓ_0, then we find from (E2) and (E3): $\omega_n/\sigma_n = n(\omega_0/\sigma_0)$ and $\ell_n/\sigma_n = n(\ell_0/\sigma_0 + 1) - 1$ for all $n \in \mathbb{Z}$. These results encompass the selection rules provided in Refs. [3, 14], and also include the "negative frequencies" for $n < 0$, which have been predicted in Li *et al.* [3] but not yet demonstrated.

Since a target with circular symmetry cannot absorb net angular momentum, we note that laser-target configurations with full rotational symmetry usually obey $\ell_A/\sigma_A = -1$ and thus exhibit "conservation of the total angular momentum"; see e.g. Refs. [3, 14, 15, 29, 30]. Conversely, a q-plate flips the polarisation of circularly polarised light and adds orbital AM to it, absorbing 2 units of AM and emitting $2q$ units [19, 20]. The total angular momentum of the beam then changes by $2(q - 1)$, which is nonzero for $q \neq 1$.

Combining all of the above, we wish to obtain a laser-plasma interaction that generates harmonics satisfying ($n \in \mathbb{Z}$, $q \in \mathbb{Q}$):

$$\omega_n/\sigma_n = n\omega/\sigma, \tag{E10}$$

$$\ell_n/\sigma_n = n(\ell/\sigma + q) - q. \tag{E11}$$

This generalises all of the above. Setting $q = 0$ returns the results in Lichters *et al.* [13]. Setting $q = 1$ returns

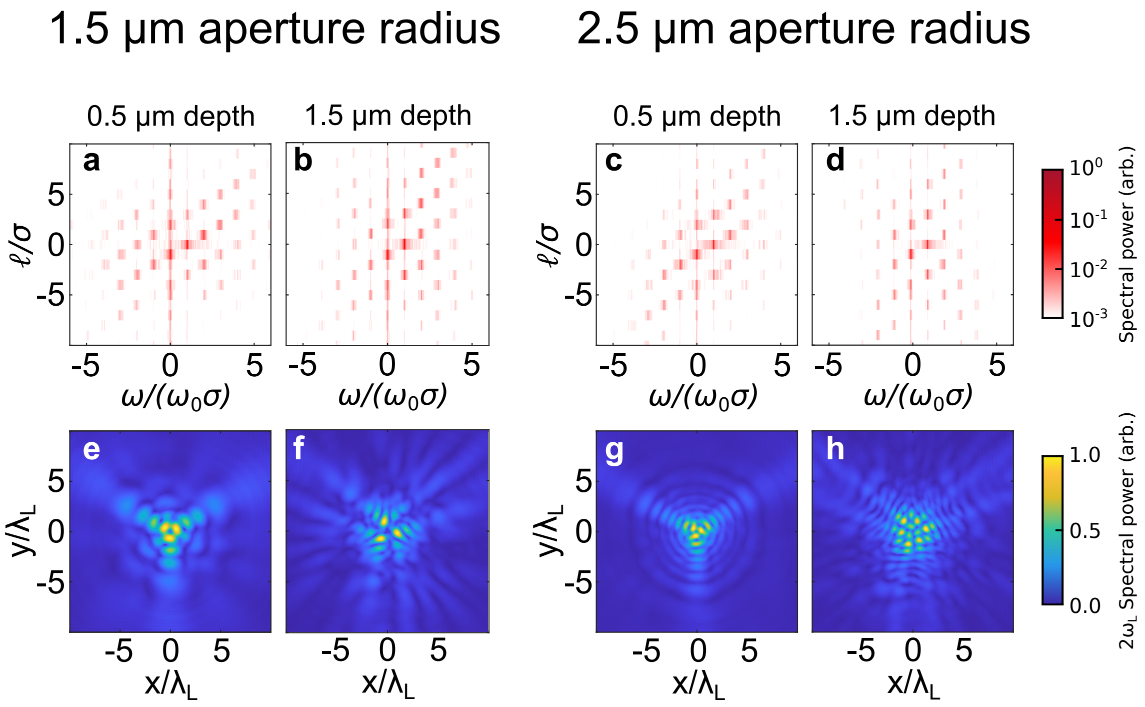

Figure S2: **a-b** 2D harmonic spectrum obtained for an aperture radius of 1.5 μm and corrugation depths of 0.5 μm and 1.5 μm, respectively. **c-d** Corresponding harmonic spectra for an aperture radius of 2.5 μm. **e-h** The 2D spatial profile of the combined $2\omega_0$ generated light for the same four preceding cases.

the results in Refs. [3, 14]. Setting $n = -1$ and thus $\sigma_n = -\sigma$ yields an ordinary q-plate [19, 20]. We note that Eqns. (E10) and (E11) correspond to Eqns. (E2) and (E3) in Section S3 with $\omega_A = 0$ and $\ell_A/\sigma_A = -q$. Thus, we need to create a DC mode with $\Psi_{DC} \propto \exp(iq\varphi)$, i.e. a shaped target surface with a height function such that $\nabla_\perp h \propto (\cos(q\varphi), \sin(q\varphi))$. To determine a suitable $h(x, y)$, we first identify its contour lines, i.e. the curves $(r(\varphi), \varphi)$ in polar coordinates for which the polar angle α of the tangent to the curve satisfies $\alpha(\varphi) = q\varphi + \alpha_0$. This leads to the following equation:

$$r'(\varphi)/r(\varphi) = \cos[\alpha(\varphi) - \varphi]/\sin[\alpha(\varphi) - \varphi]. \qquad \text{(E12)}$$

For $q \neq 1$, we can set $\alpha_0 = \pi/2$ without loss of generality, and we find $(C > 0)$:

$$r(\varphi) = C[\cos(q-1)\varphi]^{1/(q-1)}. \qquad \text{(E13)}$$

For $q = 1$ and $\alpha_0 \neq 0$, we find:

$$r(\varphi) = C\exp(a\varphi), \;\; a = \cot(\alpha_0). \qquad \text{(E14)}$$

For $q = 1$ and $\alpha_0 = 0$, we find: $\varphi = C$, r undetermined.

The next step is to determine the height function $h(x, y)$ or $h(r, \varphi)$ that we need in the laser-target interaction where we generate our harmonics. In Ref. [14], a cone-shaped dent is used with height function $h(r, \varphi) = r\tan(\pi/2 - \alpha)$, to obtain laser-plasma HG with $q = 1$. The contour lines for this shape satisfy: $z = C$, $r(\varphi) = C$, i.e Eq. (E14) with $q = 1$ and $\alpha = \pi/2$. This can easily be generalised. For $q \neq 1$, we set $h(r, \varphi) = C^{1-q}$ and invert Eq. (E13) to obtain:

$$h(r, \varphi) = r^{1-q}\cos[(q-1)\varphi]. \qquad \text{(E15)}$$

For $q > 1$, this leads to a pole at $r = 0$, so we may need to exclude the area $r < \epsilon$ for some small $\epsilon > 0$, or use e.g. $h(r, \varphi) = \arctan\{r^{1-q}\cos[(q-1)\varphi]\}$ to saturate h near $r = 0$. This is not necessarily a problem, since laser beams with nonzero OAM usually have vanishing intensity for small r, so the precise shape of the target surface near $r = 0$ is not too important. As an alternative, one can set $h = C^{q-1}$ when $q > 1$ and invert Eq. (E13) to obtain $h(r, \varphi) = r^{q-1}/\cos[(q-1)\varphi]$, and saturate h near the angles where $\cos[(q-1)\varphi] = 0$.

For $q = 1$ and $\alpha = \pi/2$, we recover the conical shape of Ref. [14]. For $q = 1$ and $\alpha = 0$, we set $h(r, \varphi) = \lambda_0\varphi/(2\pi)$ for $r \geq \epsilon > 0$ to obtain the "spiral plate" of Ref. [3]. For $q = 0$, we obtain $h(r, \varphi) = r\cos(\varphi) = x$, which corresponds to a laser beam with oblique incidence onto a plane, as described in Lichters *et al.* [13]. We see that we can design target surfaces to obtain the harmonic generation patterns dictated by Eqns. (E10) and (E11) for any q.

The salient features of the non-linear q-plate are as follows:

(i) We can have any $n \in \mathbb{Z}$, not just $n = -1$ or $n > 0$.

(ii) We can have any $q \in \mathbb{Q}$, not just $q = 0$ or $q = 1$, or (half-)integer q.

(iii) We can have $q < 0$ just as well as $q > 0$.

(iv) Surprisingly, for non-integer q, ℓ_n given by Eq. (E11) need not be an integer. However, for integer ℓ_n, the OAM spectrum for harmonic ω_n/σ_n will show a sharp peak at ℓ_n/σ_n, while for non-integer ℓ_n the OAM spectrum will show a much broader range of peaks at integer OAM levels. This distinction might allow one to select or suppress certain harmonics

(v) We note that for $J = L + S$, we find that $j_n/\sigma_n = \ell_n/\sigma_n + 1 = n(\ell/\sigma + q) - (q - 1) = n(\ell/\sigma + 1) + (n - 1)(q - 1)$, so J is conserved within the EM waves for all harmonics if and only if $q = 1$. For $q \neq 1$, total angular momentum is not conserved in the EM waves alone; the medium will have to absorb the difference.

The concept of the non-linear q-plate can be extended to that of the non-linear J-plate [21, 22]. This works as follows. A basic J-plate turns a circularly polarised beam with $\sigma = +1$ into one with $\sigma = -1$, adding ℓ_r to the OAM level. Similarly, it turns a circularly polarised beam with $\sigma = -1$ into $\sigma = +1$, adding ℓ_s to the OAM level. To ensure that ℓ_r and ℓ_s are fully independent, we need two degrees of freedom in the design of the non-linear q-plate, which we choose to be q and ℓ_J, where ℓ_J is the amount of OAM that needs to be added to the laser pulse prior to its interaction with the q-plate. We revisit Eq. (E11), with nonzero ℓ_J:

$$\ell_n/\sigma_n = n[(\ell + \ell_J)/\sigma + q] - q. \tag{E16}$$

We set $n = -1$ and use $\sigma_{-1} = -\sigma$ to obtain:

$$\ell_{-1} = \ell + \ell_J + 2q/\sigma \tag{E17}$$

For $\sigma = +1$, we find $\ell_{-1} - \ell = \ell_r = \ell_J + 2q$, while for $\sigma = -1$, we find $\ell_{-1} - \ell = \ell_s = \ell_J - 2q$. Thus, a non-linear J-plate with parameters ℓ_r, ℓ_s can be produced from an enhanced non-linear q-plate by setting $q = (\ell_r - \ell_s)/4$ and $\ell_J = (\ell_r + \ell_s)/2$.

Note that $\nabla_\perp h$ of a non-linear q-plate corresponds to the "frozen" field of a Laguerre-Gaussian mode with circular polarisation and OAM level $\ell/\sigma = -q$, see e.g. Courtial *et al.* [31]. We can exploit this to design target surfaces that correspond to e.g. the frozen field of Hermite-Gaussian modes. Irradiate this with a Hermite-Gaussian mode with linear polarisation parallel to $\nabla_\perp h$, to obtain odd harmonics with the HG mode of the pump, even harmonics with the HG mode of the target. There are many more possibilities, e.g. also exploring the radial mode of the Laguerre-Gaussian harmonics, but that is beyond the scope of this work.

S8. TRANSMISSIVE AND REFLECTIVE LASER PARAMETERS

In the main text, the laser parameters for the reflective and transmissive cases are slightly different due to originally being conducted independently to verify the harmonic predictions. The simulations for the transmission case have also been run with the same parameters as the reflective case ($I_{\max} = 6.6 \times 10^{18}$ Wcm^{-2} and 100 fs FWHM pulse duration) and the results for the n=0,2 and 5 cases are shown in Supplementary figure S3**a-c**. As can be seen, the overall harmonic spectra maintains the same profile, but the overall strength of the harmonics is reduced. This is particularly noticeable for the higher harmonics. The on-axis $y = 0$ and $x = 0$ spectra are also shown in red in Supplementary figure S3**d-f** with the original $I_{\max} = 1 \times 10^{19}$ Wcm^{-2} and 50 fs FWHM pulse duration is shown in gray for comparison. For the longer pulse, lower intensity, the $6\omega_0$ can no longer be observed above the numerical noise. In order to maintain better signal-to-noise, the higher intensity shorter pulse duration results are used throughout in the main text for the transmission cases.

[1] H. Eichmann *et al.*, Phys. Rev. A **51**, R3414 (1995).
[2] O. E. Alon, V. Averbukh and N. Moiseyev, Phys. Rev. Lett. **80**, 3743 (1998).
[3] S. Li *et al.*, New J. Phys. **22**, 013054 (2020).
[4] É. Mathieu, J. Math. Pure Appl. **13**, 137 (1868).
[5] J. Vieira *et al.*, Phys. Rev. Lett. **117**, 265001 (2016).
[6] C. Joshi, T. Tajima, J. M. Dawson, H. A. Baldis, and N. A. Ebrahim, Phys. Rev. Lett. **47**, 1285 (1981).
[7] R. Bingham, Physica Scripta **T30**, 24, (1990).
[8] R. Trines *et al.*, arXiv:2305.11659 (2023).
[9] N. Bloembergen, J. Opt. Soc. Am. **70**, 1429 (1980).
[10] K. J. Schafer *et al.*, Phys. Rev. Lett. **70**, 1599 (1993).
[11] P. B. Corkum, Phys. Rev. Lett. **71**, 1994 (1993).
[12] M. Lewenstein, *et al.*, Phys. Rev. A **49**, 2117 (1994).
[13] R. Lichters, J. Meyer-ter-Vehn, and A. Pukhov, Phys. Plasmas **3**, 3425 (1996).
[14] J. W. Wang, M. Zepf and S.G. Rykovanov, Nat. Commun. **10**, 5554 (2019).
[15] L. Yi, Phys. Rev. Lett. **126**, 134801 (2021).
[16] Y. Tang *et al.*, Nat. Photonics **14**, 658 (2020).
[17] G. Lerner *et al.*, Sci. Adv. **9**, eade0953 (2023).
[18] K. Qu, Q. Jia and N. J. Fisch, Phys. Rev. E **96**, 053207 (2017).
[19] L. Marrucci, C. Manzo and D. Paparo, Phys. Rev. Lett. **96**, 163905 (2006).
[20] L. Marrucci *et al.*, J. Opt. **13** 064001 (2011).
[21] R. C. Devlin *et al.*, Science **358**, 896 (2017).
[22] H. Sroor *et al.*, Nat. Photonics **14**, 498 (2020).
[23] D. Hickstein *et al.*, Nat. Photonics **9**, 743 (2015).
[24] L. Rego *et al.*, Sci. Adv. **8**, eabj7380 (2022).
[25] E. Pisanty *et al.*, Phys. Rev. Lett. **122**, 203201 (2019).
[26] K. M. Dorney *et al.*, Nat. Photonics **13**, 123 (2019).

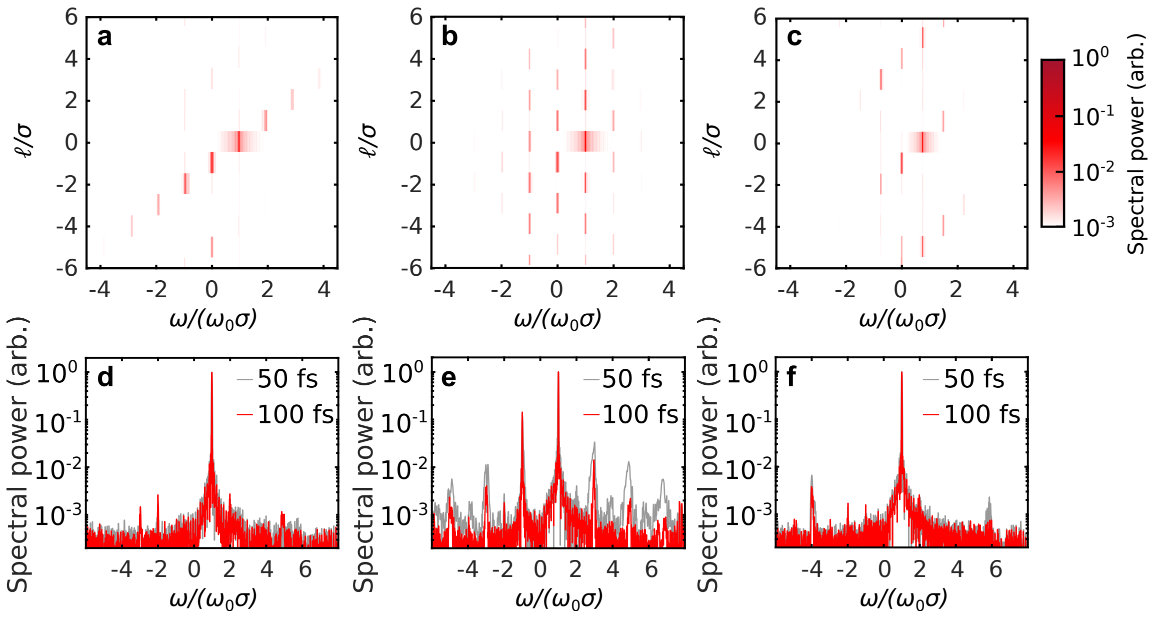

Figure S3: 2D harmonic spectrum obtained from the interaction of a CP pump pulse with $\ell = 0$ with structured aperture targets with: **a**, n=0 (q=1); **b**, n=2 (q=-1); and **c**, n=5 (q=-4), for a laser intensity of $I_{\rm max} = 6.6 \times 10^{18}$ Wcm^{-2} and 100 fs FWHM pulse duration. **d-f** Frequency spectra for the $I_{\rm max} = 6.6 \times 10^{18}$ Wcm^{-2} and 100 fs FWHM pulse duration, red, generated at $y = 0$ and $x = 0$ for the same target structures in **a-c**, respectively. The spectra for the $I_{\rm max} = 1 \times 10^{19}$ Wcm^{-2} and 50 fs FWHM pulse duration case used in the main text is shown in gray for comparison.

[27] J. F. Drake *et al.*, Phys. Fluids **17**, 778 (1974).
[28] D. W. Forslund, J. M. Kindel and E. L. Lindman, Phys. Fluids **18**, 1002 (1975).
[29] Y. Zhao *et al.*, Phys. Rev. Lett. **99**, 073901 (2007).
[30] Z. Shao *et al.*, Nat. Commun. **9**, 926 (2018).
[31] J. Courtial *et al.*, Phys. Rev. Lett. **81**, 4828 (1998).
